# Another American Dentist for Europe

**Published:** 1878-05

**Authors:** 


### ANOTHER AMERICAN DENTIST FOR EUROPE.

W. F. Thompson, M. D., who has practiced his profession in this and other cities with success and honor, took his departure for Paris in March. He holds an honorary degree from the old Ohio Dental College. Was president of the Oregon State Dental Society in 1874. He is a thorough gentleman, generous in every particular, is a number one operator, but his health was not good here. A host of D. D. S.'s wish him a warm reception wherever he visits or locates. W.

San Francisco, April 2, 1877.

